# Gynecology

**Published:** 1875-11

**Authors:** 


### II. Gynecology.

1. *Methods of rendering the Female Urinary Bladder accessible, and on Probing the Ureter in Woman.* Prof. Simon, of Heidelberg. Translated by Aug. C. Bernoys, M.D. (*N. Y. Med. Jour.*, October, 1875.)

The professor enlarges upon and explains his methods, first communicated to the public by his pupil, Wildt, in the *Archives of Clinical Surgery* (v. 18, p. 167).

The methods heretofore pursued are (*a*) bloodless dilatation of the urethra, (*b*) urethrotomy, (*c*) vagino-vesical section, (*d*) vestibular section, and (*c*) the suprapubic stone operation. The first and third methods will probably alone survive for removal of stone and effective local treatment in other diseases. In the first method, gradual will have to yield to rapid dilatation.

The proposed exploratory method consists of three acts, viz: the slitting of the urethral orifice, the dilatation of the urethra itself by plug-shaped specula, and the subsequent bimanual digital palpation of the bladder. It is called bloodless, because, although small splits are made at the edge of the orifice, the dilatation of the urethra itself is accomplished without loss of blood.

Two lateral incisions of ¼ ctm. are made in the upper margin, and one downward of ½ ctm. in depth. If needful the stretched tissue is easily split further by using speculum or finger as conductor. In consequence, the finger penetrates more deeply: that is from ¼ to ½ of a centimetre, *to which extent the urethra has been shortened.* Incontinence does not result, as the muscular fibres are undivided. This slitting is done best with scissors. In the second act, the smooth plugs are far preferable to the wrinkled finger. They are made of hard rubber, cut off straight at the point, and shutting with a rounded mandrin. Seven sizes are used: the smallest ¾, the largest 2 centimetres in diameter. They are also specula. After them follows the finger.

On passingthe forefinger, (for which no rotatory motion (Heath) is requisite), the middle finger of the same hand is introduced into the vagina; and the two advanced until the margin of the urethro-vaginal septum presses against the digital commissure. The middle finger, if doubled into the hand, will impinge upon the labia, and advance will be retarded by one centimetre. The vesical apex is then pressed against the exploring finger, with the other hand. Its inverted mucous surface is thus directly explored by the finger.

Dilatation may be effected with plugs of 1.9 to 2 centimetres in diameter (6 to 6.3 in circumference) without danger of resulting incontinence in adults. The operator's finger measures at base not quite 6 centimetres in circumference (1.8 in diameter). By the side of this, in three instances, he has introduced spoons and fingers into the bladder—the stems lying in the urethra next to the finger. The circumference was thus increased to 6.5 and 6.8, and in changed positions it amounted to, at the most, 7 centimetres. In girls, 4.7 to 6.3 centimetres in circumference, are the measures inside of which the surgeon should confine himself.

The professor states that after convincing himself of the perfect safety of the performance, he did not think it wrong to make the exploration in such women as volunteered for the purpose ; and that he has practiced digital palpation in over sixty cases in Heidelberg during the last two years and a half. The profession at large will hardly assent to the propriety of such expedients in any case when entirely needless.

Vesico-vaginal section is performed by making a *transverse* incision, 3 ctm. in length, into the anterior vault of the vagina, one-fourth to one-half ctm. in front of the anterior lip of the os uteri. By *this means the bladder is introverted through the incision into the vagina, and even into the vulva itself.* Occasionally the opening was made T shaped, by a second incision carried at right angles toward the urethra. Introversion was effected by a fine double hook, inserted into the vesical mucous lining, with conjoined pressure from above. Hæmorrhage is stopped by torsion or ligature. The bladder is thus made so completely accessible, that the most complicated and difficult operations are performed with as much facility as if on the surface of the body.

Subjoined are the indications for the operation, space forbidding the author's comments on each :

1. Diseases of the mucous membrane requiring diagnosis. 2. Stones and foreign bodies, (diagnosis and extraction). 3. Cauterization for inveterate vesical catarrh. 4. Urethral fissures. 5. In kolpo-kleisis with defect of vesico-vaginal septum. 6. Diagnosis of seat and extent of growths and tumors in that septum. 7. Extirpation of tumors (especially papillomata) from vesical membrane. 8. Discovery, extraction or excision of renal calculi, from the vesical part of the ureter. 9. Opening of hæmatometra, when puncture is impossible or too dangerous between the bladder and rectum. 10. Cure of colo-vesical or entero-vesical fistula, by cauterizing the vesical orifice of the fistula.

Subjoined are the indications for kolpo-cystotomy :— 1. Large stones with great sensibility of bladder. 2. Production of direct escape of urine, in inveterate vesical catarrh, with ulcerations of mucous lining. 3. Extirpation of tumors and excrescences, situated so high in the lateral parts of the bladder, that they cannot be made directly accessible through the dilated urethra alone. 4. Cure of colo-vesical or entero-vesical fistulæ, incurable by cauterization after urethral dilatation.

*The probing and catheterization of the ureter from the bladder*, after the dilatation of the urethra described above, is a procedure the credit of which Professor Simon can alone claim. At the risk of unduly prolonging this abstract, we give the *ipsissima verba* of the translation :

"After the urethra is dilated in the above-described way, we search for the ligamentum interuretericum with the finger. This ligament is about one inch from the sharply-marked internal orifice of the urethra ; in the middle it is usually so little prominent that it can only be distinguished by experienced explorers. Around the orifice of the ureter, which is one-half to three-quarters of an inch away from the middle of this ligament, the muscular coat of the ureter, which ends in the interureteric ligament, forms a kind of a pad, and is easy to distinguish. The orifices on these pads are very thin slits, and, since they have only very narrow edges of mucous membrane, they are imperceptible to the touch. On account of this, the third act, viz., the introduction of the probe, is rendered more difficult. In order to effect it we must fix the "Harnleiterwulst" (the vesical fold where the orifice lies) with the finger in that region where the orifice must be situated, and then push the head of the probe, which lies close to the side of the finger, toward this region in the direction of the ligamentum interureterícum from the inside and below, upward and outward. The handle of the instrument must be led to the opposite side and at the same time be raised up against the arcus pubis, in order that the head of it may not glide off from the very steep trigonum. By slightly pushing we try to introduce the head of the probe into the orifice of the ureter. If the probe does not go into the orifice, it will be arrested by the walls of the bladder; but if it enters, it can easily be pushed on in an upward and outward direction. The inlying finger tells whether the probe has remained in the cavum vesicæ, or whether it has really entered into the orifice. In the latter case, we feel the probe covered by mucous membrane for a few centimetres, and we can feel the borders of the orifice all around the probe. If we wish to sound the pelvis of the kidney, we have only to push the probe on in a lateral direction until at a height of seven to eight centimetres, and we strike the brim of the true pelvis (linea innominata). Now it becomes necessary to move the handle of the probe to the inner face of the thigh of that side on which the ureter is probed, and to incline it so that the inner end of it is placed parallel to the vertebral column, and the head directed more toward the anterior abdominal coverings. In this direction the probe advances very readily into the upper end of the ureter and the pelvis of the kidney. If the catheter has been used instead of the probe, the urine will now ooze out drop by drop, or sometimes it will spurt out in a stream at intervals of a half to one minute."

The probe and catheter used are as thick as common fistula probes, and are 25 centimetres in length. The handles are movable, and fastened with a screw to aid in lengthening or shortening them. The metal used in their construction should not be too flexible. Elastic instruments are proscribed. The probes are more easy of introduction than catheters.

The process may prove useful in nephro-lithiasis, and in diagnosis of unilateral renal disease, being an improvement upon the method of Tuchmann of London.

The professor has now employed this method in about twenty-four cases.

2. *Hysterotomy in the Treatment of Uterine Fibroid Tumors.* Pozzi, S. (Reviewed in *Le Progrès Médic.*, Aug. 14.)

The following are the author's conclusions :

1. Abdominal hysterotomy in the treatment of fibroid tumors of the uterus, is, though yet quite novel, perfectly justifiable in many cases, and deserves a definite rank among the operations of surgery.

2. There is no comparison between the indications for gastrotomy in cases of fibroid uterine tumors, and for ovarian cystic disease. The fatal tendency in the latter class of diseases, calls for and justifies an operation in the larger number of cases, which is not indicated in the immense majority of large fibroid tumors.

3. The operation should be reserved for fibroid, or fibro-cystic tumors of rapid or excessively rapid evolution, and accompanied by dangerous symptoms.

4. The fibroid tumors, which are larger than those referred to above, even when they produce alarming symptoms, require less heroic treatment. Their natural tendency to decrease in size or to establish toleration of their presence, is well recognized. Experience has demonstrated that an expectant treatment in these cases, is followed by less mortality than operations for hysterotomy.

5. When, in consequence of an error in diagnosis, gastrotomy discloses a uterine tumor, and not an ovarian cyst, ablation is preferable to leaving the operation unfinished, even though the fibroid tumor be not pediculated.

Under the head of cervical hysterotomy, the author includes simple division of the neck; incision followed by enucleation where the tumor is developed in the tissue of the cervix; and lastly, complete amputation of the cervix when it encloses in its substance a largely developed fibroid tumor productive of true hypertrophic elongation.

Intrauterine hysterotomy first described by Velpeau, first practiced by Amussat, finding but little favor with the French and enthusiastically adopted by the English and Americans, is described according to the procedure of Marion Sims.

